# Supplementary material for: Tuning quantum measurements to control chaos
Source: Sci Rep. 2017 Mar 20;7:44684. doi: 10.1038/srep44684 (PMC5357801; doi:10.1038/srep44684)
Supplement: Supplementary Information [file srep44684-s1.pdf]

Tuning quantum measurements to control chaos: Supplemental Material

Jessica K. Eastman[1,*], Joseph J. Hope[2], and André R. R. Carvalho[1]

[1]Centre for Quantum Computation and Communication Technology, Department of Quantum Science, Research School of Physics and Engineering, The Australian National University, Canberra ACT 2601 Australia
[2]Department of Quantum Science, Research School of Physics and Engineering, The Australian National University, Canberra ACT 2601 Australia
[*]jessica.eastman@anu.edu.au

Supplementary video 1

Animation of the evolution of the Wigner function for the full dynamics for a single trajectory with the parameters $|u| = 1$, $\beta = 0.3$, $\Gamma = 0.10$, $g = 0.3$, $\Omega = 1$ and $\phi = \pi/2$. The basis size for the numerical calculation is $N = 65$. For these parameters, the classical system is chaotic.

Supplementary video 2

Animation of the evolution of the Wigner function for the full dynamics for a single trajectory with the parameters $|u| = 1$, $\beta = 0.3$, $\Gamma = 0.10$, $g = 0.3$, $\Omega = 1$ and $\phi = \pi$. The basis size for the numerical calculation is $N = 65$. For these parameters, the classical system is chaotic.

Supplementary video 3

Animation of the evolution of the Wigner function for a single trajectory of a horizontal CAT state undergoing continuous monitoring with $\phi = \pi$.

Supplementary video 4

Animation of the evolution of the Wigner function for a single trajectory of a horizontal CAT state undergoing continuous monitoring with $\phi = \pi/2$.

Supplementary video 5

Animation of the evolution of the Wigner function for the full dynamics for a single trajectory with the parameters $|u| = 1$, $\beta = 0.3$, $\Gamma = 0.05$, $g = 0.3$, $\Omega = 1$ and $\phi = \pi$. The basis size for the numerical calculation is $N = 65$. For these parameters, the classical system is regular.

Supplementary video 6

Animation of the evolution of the Wigner function for the full dynamics for a single trajectory with the parameters $|u| = 1$, $\beta = 0.3$, $\Gamma = 0.05$, $g = 0.3$, $\Omega = 1$ and $\phi = \pi/2$. The basis size for the numerical calculation is $N = 65$. For these parameters, the classical system is regular.
